# An Improved High-Sensitivity Airborne Transient Electromagnetic Sensor for Deep Penetration

**DOI:** 10.3390/s17010169

**Published:** 2017-01-17

**Authors:** Shudong Chen, Shuxu Guo, Haofeng Wang, Miao He, Xiaoyan Liu, Yu Qiu, Shuang Zhang, Zhiwen Yuan, Haiyang Zhang, Dong Fang, Jun Zhu

**Affiliations:** 1 College of Electronic Science and Engineering, Jilin University, Changchun 130012, China; chenshudong@jlu.edu.cn (S.C.); guosx@jlu.edu.cn (S.G.); whf@jlu.edu.cn (H.W.); evergreen92@163.com (M.H.); 18204319478@163.com (X.L.); 18443143119@163.com (Y.Q.); 2 Science and Technology on Near-Surface Detection Laboratory, Wuxi 214035, China; yuanzw2008@126.com (Z.Y.); zhyzhyzhy001@126.com (H.Z.); csdfangdong@163.com (D.F.)

**Keywords:** transient electromagnetic sensor, deep penetration, sensor internal noise, EMC design

## Abstract

The investigation depth of transient electromagnetic sensors can be effectively increased by reducing the system noise, which is mainly composed of sensor internal noise, electromagnetic interference (EMI), and environmental noise, etc. A high-sensitivity airborne transient electromagnetic (AEM) sensor with low sensor internal noise and good shielding effectiveness is of great importance for deep penetration. In this article, the design and optimization of such an AEM sensor is described in detail. To reduce sensor internal noise, a noise model with both a damping resistor and a preamplifier is established and analyzed. The results indicate that a sensor with a large diameter, low resonant frequency, and low sampling rate will have lower sensor internal noise. To improve the electromagnetic compatibility of the sensor, an electromagnetic shielding model for a central-tapped coil is established and discussed in detail. Previous studies have shown that unclosed shields with multiple layers and center grounding can effectively suppress EMI and eddy currents. According to these studies, an improved differential AEM sensor is constructed with a diameter, resultant effective area, resonant frequency, and normalized equivalent input noise of 1.1 m, 114 m^2^, 35.6 kHz, and 13.3 nV/m^2^, respectively. The accuracy of the noise model and the shielding effectiveness of the sensor have been verified experimentally. The results show a good agreement between calculated and measured results for the sensor internal noise. Additionally, over 20 dB shielding effectiveness is achieved in a complex electromagnetic environment. All of these results show a great improvement in sensor internal noise and shielding effectiveness.

## 1. Introduction

Over the last two decades, airborne transient electromagnetic (AEM) systems have become increasingly popular for hydrogeophysical investigations, engineering surveys, unexploded ordnance (UXO) detection, as well as geological mapping and mineral exploration [1,2,3]. The development of the AEM system has resulted in more accurate penetration with a greater depth [4,5]. However, further improvement in penetration depth requires more work. In this article, we will discuss the design and optimization of a high-sensitivity AEM sensor for deep penetration.

According to Spies, the investigation depth of transient electromagnetic (TEM) is proportional to the inverse fifth power of the system noise [6], which is mainly composed of sensor internal noise, electromagnetic interference, environmental noise, etc. Thus, an improved high sensitivity AEM sensor with low sensor internal noise and good shielding effectiveness is of great significance for deep penetration.

As one of the core components of the AEM system, the sensor consists of two parts: an induction coil and a pre-amplifier. The induction coil transfers the changes in the magnetic flux into a voltage proportional to the rate of change [7]. A pre-amplifier is used to amplify the weak signals induced in the coil and, therefore, plays an important role in retarding the degrading of the signal to noise ratio (SNR) [8]. As one of the most important parameters of the sensor, sensitivity mainly depends on the sensor internal noise and electromagnetic compatibility, both of which will be discussed in this article.

The sensor internal noise represents the limitation of the system noise, which has been addressed in the published literature. Dehmel has discussed the influence of the number of turns n on the noise level of an air-core coil within weight constraints [9]. Tumanski et al. calculated parameters, such as the resultant area, direct current (DC) resistance, and SNR of an induction coil [10]. However, they considered thermal noise of the coil’s DC resistor as the only noise source and did not take into account the preamplifier noise, which, in fact, plays an important role in the sensor internal noise. The noise model with a preamplifier has been constructed for induction coil magnetometers by researchers such as Séran et al. [11] and Lin et al. [12]. These studies show that the total noise of the sensor is mainly composed of three parts: the thermal noise of the coil resistor, and voltage, and the current noise of the amplifier. For the purpose of improving the performance of an air-coil sensor suited to helicopter TEM exploration, the sensor internal noise model with both an induction coil and preamplifier is investigated by Chen [13,14]. However, optimization of the induction coil is carried out without taking into account the noise from the preamplifier and the damping resistor, which, in fact, are responsible for most of the sensor internal noise.

Electromagnetic compatibility (EMC) design is essential for the sensitivity of the sensor, especially for systems working in complicated electromagnetic environments, such as the AEM system. A center-tapped air-core coil combined with a differential pre-amplifier is chosen to suppress the common-mode noise induced in exploration surveys [13,14]. Electromagnetic shielding, which has been widely used in search coil magnetometers [15] and quantum magnetometers [16], has also been used in TEM sensors. For example, a new shielded receiver coil with a noise level 2–3 times less than the conventional one has been developed by SkyTEM [17]. However, problems such as shielding effectiveness optimization, eddy current suppression, and shield grounding are rarely discussed in detail. These problems are crucial to the performance of the sensor, especially for systems working in complex electromagnetic environments.

An improved differential AEM sensor with triple shielding for deep penetration is proposed here. The rest of the paper is organized as follows. With the sensor internal noise level determined by penetration depth, the noise model with both induction coil and preamplifier is established and analyzed first. Then, the influence of the technical specifications such as diameter, resonant frequency, and bandwidth of the system is analyzed. Then, the EMC design of the sensor shielding is discussed in detail. Finally, experiments are conducted to verify the accuracy of the sensor internal noise model and electromagnetic compatibility of the sensor.

## 2. Sensor Internal Noise Determination

As an important parameter, sensor internal noise has considerable influence on the performance of the system. According to Spies, the penetration depth *H* depends on the noise level *V_n_* of the system, peak moments *M* of transmitting current and ground resistivity *ρ_e_* [6]:

(1) $$H=0.55{\left(M\rho_{e}/V_{n}\right)}^{1/5}$$

As mentioned above, the system noise *V_n_* is composed of sensor internal noise, environmental noise, and electromagnetic interference. Sensor internal noise is the only one that determines the maximum penetration depth of the system. In actual exploration, the detection error caused by the sensor internal noise *V_ns_* should not exceed 2% [11]. That is:

(2) $$V_{ns}\leq 0.02V_{n}$$

**Figure 1:**
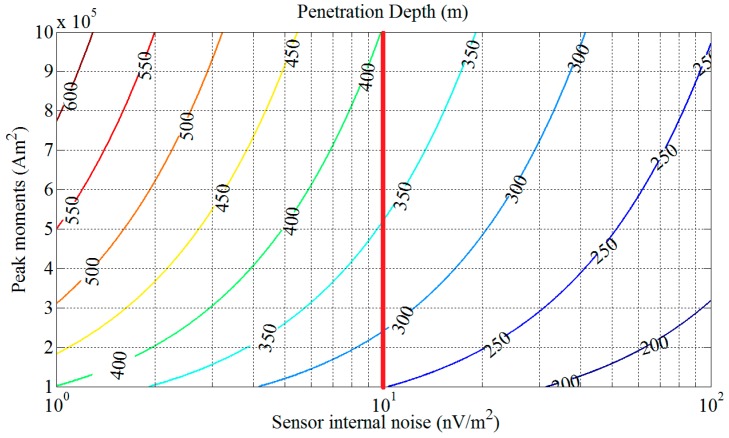
Penetration depth *H* versus sensor internal noise *V_ns_* and peak moments *M*.

According to Equations (1) and (2), if the Earth’s electrical conductivity *ρ_e_* in the half-space model is generally set to 0.01 S/m, the penetration depth *H* versus sensor internal noise *V_ns_* and peak moment *M* of transmitting current is shown in Figure 1.

As shown in Figure 1, if the peak moment *M* is set to 10^5^–10^6^ Am^2^, and the sensor internal noise is set to 1–100 nV/m^2^, the corresponding penetration depth is then found to range from 200 m to 600 m. When sensor internal noise is chosen as 10 nV/m^2^, the corresponding penetration depth changes from 250 m to 400 m, with the peak moments increasing from 10^5^ Am^2^ to 10^6^ Am^2^, as indicated by the red line in Figure 1.

Based on the discussion above, a sensor internal noise level of approximately 10 nV/m^2^ is determined for the AEM sensor. The detailed process of design and optimization of the sensor with this goal will be described in the following sections. We will first present the physical structure and equivalent electrical model of the sensor in Section 3.

## 3. Structure and Equivalent Electrical Model of the Induction Coil

In this section, the physical structure of a shielded differential induction coil will be illustrated, followed by the equivalent electrical model of the coil. Then, the electrical parameters will be calculated with the geometrical parameters of the coil for further study.

### 3.1. Structure and Electrical Model of the Coil

**Figure 2:**
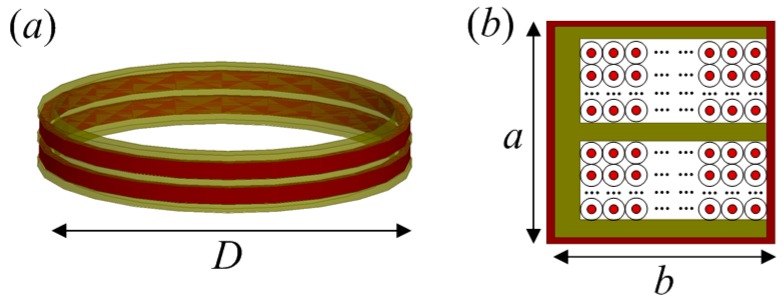
(**a**) Induction coil with differential structure; and (**b**) cross-section of the coil with shield.

The differential AEM sensor consisting of two coils that are connected in series is shown in Figure 2.

As shown in Figure 2, *D*, *a*, and *b* are the diameter of the induction coil, the height of the coil section, and the width of the coil section, respectively. The yellow part in the cross-section is the framework of coil and the peripheral part of the cross-section is the coil shield.

**Figure 3:**
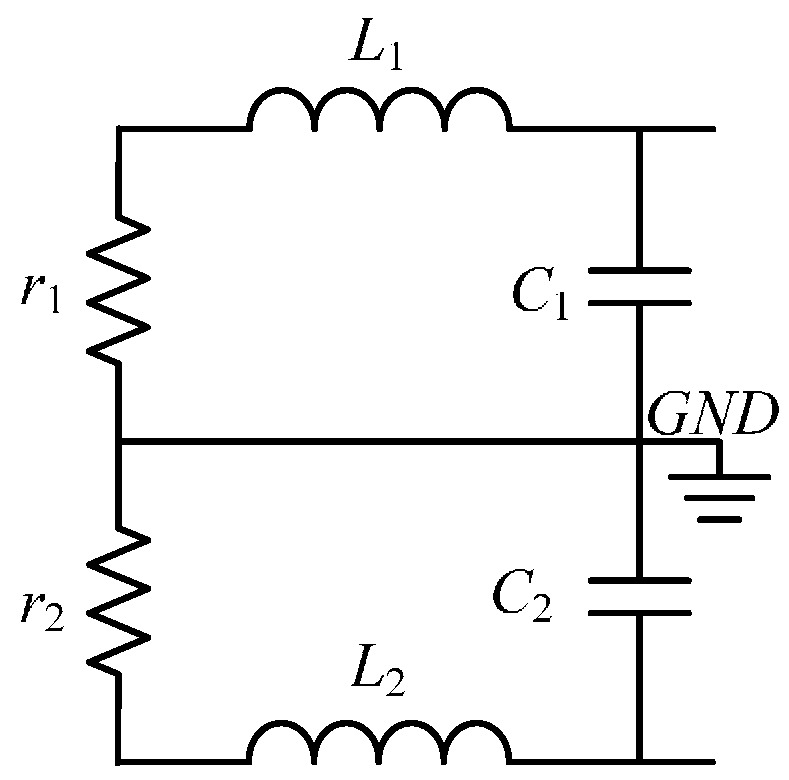
Schematic of the air-core coil.

The equivalent electrical model of the differential induction coil described in Figure 2 is shown in Figure 3.

In Figure 3, *L*_1_ = *L*_2_, *r*_1_ = *r*_2_, *C*_1_ = *C*_2_ are the inductance, resistance, and the capacitance of the coil, respectively. Of all the parameters in Figure 3, the resistance and inductance can be well predicted based on the geometrical parameters. The stray capacitance which results from several electrical couplings is difficult to estimate. The following section provides an estimate of the electrical parameters of the coil.

### 3.2. Electrical Parameters Estimation

We will estimate all the parameters in Figure 3. Additionally, parameters, such as resultant area *S* and resonant frequency *f*_0_, are also calculated. All of the estimates are based on the assumption that the diameter *D* of the coil is far greater than the cross-sectional dimensions *a* and *b*.

(a) Estimation of the coil’s DC resistance
  The coil’s DC resistance *r*_1_ is given by:
  (3) $$r_{1}=\frac{\rho Dn\pi }{2S_{c}}$$
  Here, *ρ* is the electrical resistivity of wire, *n* is the number of turns, and *S*_c_ is the area of the copper wire.
(b) Determination of the coil’s self-inductance
  For an air-core induction coil, when the diameter *D* is far greater than the cross-sectional dimensions *a* and *b*, we estimate the inductance using the following expression [18]:
  (4) $$L_{1}=\frac{\mu_{0}Dn^{2}}{4}\left[\ln\left(\frac{8D}{l}\right)-1.75\right]$$
  Here, *μ*_0_ is the permeability of vacuum, and *l* is the equivalent radius of coil section.
(c) Determination of the coil’s capacitance
  The estimation of the coil’s capacitance is quite difficult. For a rough calculation, we suppose that the total capacitance can be divided into three kinds: coil self-capacitance *C_A_*, section-to-section capacitance *C_B_*, and shield-to-coil capacitance *C_C_*. We assume that every section is composed of *n_a_* layers, and there are *n_b_* turns in each layer. The distance between layers is equal to the diameter of the wire. According to Seran [11], the coil self-capacitance *C_A_* and the section-to-section capacitance *C_B_* can be expressed as follows:
  (5) $$C_{A}=\frac{\varepsilon_{0}\varepsilon_{r}n_{b}\pi D}{2(n_{a}-1)}$$
  (6) $$C_{B}=\frac{\varepsilon_{0}\varepsilon_{r1}D\pi b}{4w}$$
  Here *ε*_0_ is the permittivity of vacuum, *ε_r_* is the relative permittivity of the wire, *ε_r_*_1_ is the relative permittivity of the framework, and *w* is the distance between two sections.
  When the shielding is wrapped around the coil, the stray capacitance between the coil and the shield will reach dozens or even hundreds of pF. The estimation of such a capacitance is quite difficult. No precise mathematical expression has been formulated so far.
  The capacitance *C*_1_ in Figure 3 can be calculated as follows:
  (7) $$C_{1}=2\left(C_{A}+C_{B}+C_{C}\right)$$
(d) Determination of the coil’s resultant area
  The coil’s resultant area can be calculated as follows:
  (8) $$S=\frac{D^{2}n\pi }{4}$$
(e) Resonant frequency of the coil *f*_0_
  The resonant frequency is determined by the inductance and stray capacitance of the coil. The resonant frequency of the coil is given by:
  (9) $$f_{0}=\frac{1}{2\pi \sqrt{L_{1}C_{1}}}$$
  Based on the electrical model and the parameters calculated above, the sensor internal noise model will be established and the optimization for the sensor parameters will be described in the next section.

## 4. Noise Model of the Sensor

In this section, the sensor internal noise model will be established and discussed in detail. According to the discussion, we attempt to find the relationship between the sensor internal noise level and parameters. Then, the optimization for the sensor parameters is carried out with these relationships.

### 4.1. Sensor Internal Noise Model

**Figure 4:**
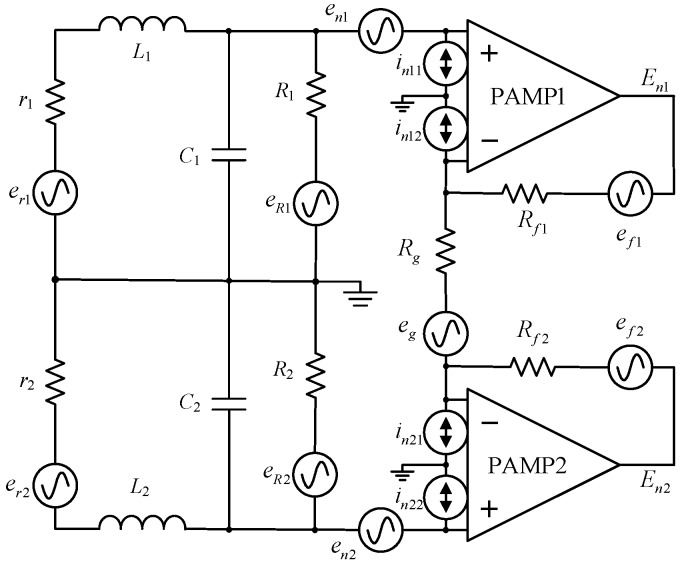
Sensor circuit with equivalent noise locations.

A central-tapped air-core coil combined with a differential pre-amplifier is chosen to suppress the common-mode noise induced in exploration surveys. The calculation of the noise level at the output of the preamplifier takes into account the different noise sources represented in Figure 4.

As seen in Figure 4, *r*_1_, *r*_2_, *L*_1_, *L*_2_, *C*_1_, *C*_2_, *R*_1_, and *R*_2_ are the DC resistance, self-inductance, distributed capacitance, and damping resistance of the induction coil in differential model. *R_g_*, *R_f_*_1_, and *R_f_*_2_ are gain resistance and feedback resistance of the preamplifier. We will calculate each noise contribution at outputs *E_n_*_1_ and *E_n_*_2_.

The total output noise of the sensor *E_n_*_1_, *E_n_*_2_ consists of three components: the thermal noise of the resistors, and the voltage and current noise of the preamplifier. The detailed calculation is shown in the following section.

### 4.2. Sensor Internal Noise Calculation

The thermal noise power spectral density (PSD) of a resistor is 4*kTr*, where *k* is Boltzmann’s constant, *T* is the absolute temperature, and *r* is the resistance. Thus, the thermal noise PSD at the output of the preamplifier is calculated for the resistors *r*_1_, *R*_1_, *R_g_*, and *R_f_*:

(10) $$E_{nr_{1}}^{2}=\frac{e_{r_{1}}^{2}\omega_{0}^{4}G^{2}}{{\left(\omega^{2}+\omega_{p}^{2}\right)}^{2}}$$

(11) $$E_{nR_{1}}^{2}=\frac{e_{R_{1}}^{2}\omega_{0}^{4}(\omega^{2}L_{1}^{2}+r_{1}^{2})G^{2}}{R_{1}^{2}{\left(\omega^{2}+\omega_{p}^{2}\right)}^{2}}$$

(12) $$E_{nR_{g}}^{2}=G^{2}e_{R_{g}}^{2}$$

(13) $$E_{nR_{f_{1}}}^{2}=e_{R_{f_{1}}}^{2}$$

Here, *G* = (2*R_f_*_1_**/***R_g_*) + 1 is the gain of the preamplifier and *ω*_0_ = 2π*f*_0_ is the resonant frequency of the coil, $\omega_{p}=\omega_{0}\sqrt{r_{1}R_{1}}$.

The output voltage noise PSD $E_{ne_{1}}^{2}$ of the preamplifier is given as:

(14) $$E_{ne_{1}}^{2}=G^{2}e_{n1}^{2}$$

The preamplifier current noise produces a voltage noise at the output of the preamplifier:

(15) $$E_{ni_{11}}^{2}=\frac{\omega_{0}^{4}(\omega^{2}L^{2}+r^{2})G^{2}i_{n11}^{2}}{{\left(\omega^{2}+\omega_{p}^{2}\right)}^{2}}$$

(16) $$E_{ni_{12}}^{2}=i_{n12}^{2}R_{f1}^{2}$$

Assuming uncorrelated noise sources, the total noise PSD is:

(17) $$E_{n}^{2}=2E_{nr_{1}}^{2}+2E_{nR_{1}}^{2}+E_{nR_{g}}^{2}+2E_{nR_{f_{1}}}^{2}+2E_{ne_{1}}^{2}+2E_{ni_{11}}^{2}+2E_{ni_{12}}^{2}$$

According to Equations (10)–(17), the equivalent noise PSD at the preamplifier input can be obtained by normalizing the output PSD of the amplifier:

(18) $$\frac{E_{n}^{2}}{G^{2}}=\frac{2\omega_{0}^{4}}{{\left(\omega^{2}+\omega_{p}^{2}\right)}^{2}}\left[e_{r_{1}}^{2}+\left(\frac{e_{R_{1}}^{2}}{R_{1}^{2}}+i_{n_{11}}^{2}\right)\left(\omega^{2}L_{1}^{2}+r_{1}^{2}\right)\right]+2e_{n1}^{2}+e_{R_{g}}^{2}+\frac{2}{G^{2}}\left(e_{R_{f_{1}}}^{2}+i_{n_{11}}^{2}R_{f_{1}}^{2}\right)$$

As can be seen in Equation (18), the equivalent noise PSD of the sensor depends on a variety of factors. Therefore, it is very difficult to analyze and optimize directly. It is necessary to simplify Equation (18) for the optimization of the sensor’s parameters.

### 4.3. Noise Simplification

From Equation (18), the equivalent noise PSD of the preamplifier has its origins in a variety of noise sources. The contributions of each noise source to the total noise PSD are different. Equation (18) is simplified to find the dominant noise source of the total noise PSD. Thus, an estimate for all noise PSD at the resonant frequency is given in Table 1.

As shown in Table 1, compared with the damping resistor *R*_1_, the normalized noise of the DC resistor *r*_1_, gain resistor *R_g_*, and feedback resistor *R_f_*_1_ can be ignored. With the development of the low-noise preamplifier, the voltage noise and current noise of the preamplifier will continue to decrease (e.g., fA/$\sqrt{\mathrm{Hz}}$ current noise, less than 1 nV/$\sqrt{\mathrm{Hz}}$ voltage noise). Therefore, the noise of the preamplifier can also be neglected.

According to the above discussion, the thermal noise of the damping resistor dominates the total noise PSD at the input of the preamplifier. Therefore, the thermal noise of the damping resistor will be considered as the only source of noise here. In this case, Equation (18) can be simplified as:

(19) $$\frac{E_{n}^{2}}{G^{2}}\approx \frac{2\omega_{0}^{4}}{{\left(\omega^{2}+\omega_{p}^{2}\right)}^{2}}\frac{\left(\omega^{2}L_{1}^{2}+r_{1}^{2}\right)e_{R_{1}}^{2}}{R_{1}^{2}}$$

The equivalent input noise power of the amplifier $V_{n}^{2}$ can be obtained by integrating Equation (19) in the range [0, BW], where BW is the bandwidth of the system set as *pf*_0_. Parameter *p* is the ratio of BW to *f*_0_ and *p* > 0.

(20) $$V_{n}^{2}=\int_{0}^{BW}\frac{E_{n}^{2}}{G^{2}}df\approx \frac{8kT}{\pi C_{1}}\arctan(p)+4kTr_{1}f_{0}\left(\frac{r_{1}}{R_{1}}-\frac{4R_{1}}{r_{1}}\right)\left(\frac{p}{p^{2}+1}+\arctan(p)\right)$$

As seen from Table 1, the DC resistance *r*_1_ is much less than the damping resistance *R*_1_, so the *r*_1_/*R*_1_ term in Equation (20) can be ignored.

The damping resistor *R*_1_ is given as:

(21) $$R_{1}=\frac{-r_{1}+2\sqrt{L_{1}/C_{1}}}{4-r_{1}^{2}C_{1}/L_{1}}\approx \frac{L_{1}\omega_{0}}{2}$$

According to Equations (20) and (21) this can be simplified as:

(22) $$V_{n}^{2}\approx \frac{4kT}{\pi C_{1}}\left(\arctan(p)-\frac{p}{p^{2}+1}\right)$$

According to Equations (4), (8), (9) and (22), the normalized noise to the resultant area *S* of the coil is given as follows:

(23) $$V_{nS}=\frac{\sqrt{V_{n}^{2}}}{S}=\frac{8\sqrt{\mu_{0}kT}}{\sqrt{\pi }}\frac{f_{0}\sqrt{\ln\left(8D/l\right)-1.75}}{D^{3/2}}\sqrt{\arctan(p)-\frac{p}{p^{2}+1}}$$

As shown in Equation (23), sensor internal noise *V_nS_* depends on four factors: the coil resonance frequency *f*_0_, the coil diameter *D*, the equivalent radius *l* of the coil’s cross-section, and the bandwidth *pf*_0_ of the system.

With the sensor internal noise set as 10 nV/m^2^, the determination of the four parameters will be realized one by one according to Equation (23).

### 4.4. Determination of the Sensor Parameters

By setting the two of the four parameters, the contour map of the normalized background noise versus the remaining two is made. According to the contour map, we approximately determine the range of the two parameters. The final decision of the parameters will depend on many other factors, such as the size and resonant frequency limitation.

#### 4.4.1. Determination of Diameter and Resonant Frequency

**Figure 5:**
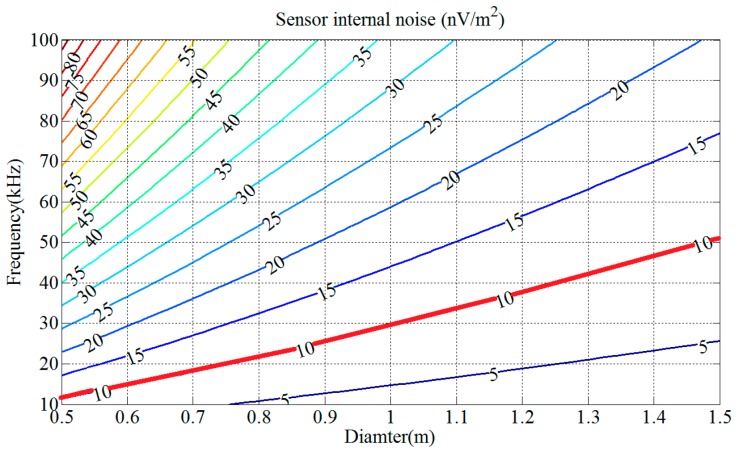
Contour map of the normalized sensor internal noise *V_nS_* versus diameter *D* and resonant frequency *f*_0_.

By Equation (23), the contour map of sensor internal noise *V_nS_* is shown in Figure 5, in which the value of *V_nS_* depends on those of *D* and *f*_0_ when *l* = 23 mm and *p* = 1.

As shown in Figure 5, the sensor internal noise *V_nS_* decreases rapidly with the diameter *D* and increases rapidly with the resonant frequency *f*_0_. The red line in Figure 5 represents the sensor internal noise V*_nS_* set as 10 nV/m^2^. Coil diameter *D* and resonant frequency *f*_0_ corresponding to the area below the red line satisfy the conditions, which means that a larger diameter with a lower resonant frequency can achieve lower sensor internal noise.

For deep penetration, the resonant frequency *f*_0_ of the sensor will be set between 30 kHz and 40 kHz and the corresponding diameter *D* changes from 1.0 m to 1.2 m. Finally, the diameter and resonant frequency of the coil are designed as 1.1 m and 35.6 kHz.

#### 4.4.2. Determination of Cross-Section Radius

**Figure 6:**
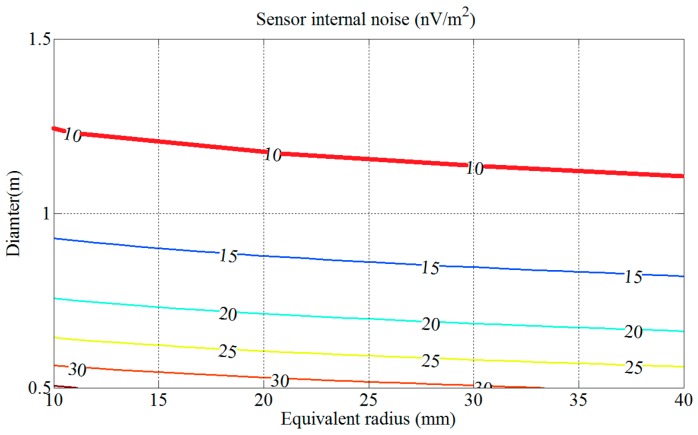
Contour map of the normalized sensor internal noise *V_nS_* versus diameter *D* and radius *l*.

By Equation (23), the contour map of the sensor internal noise *V_nS_* is shown in Figure 6, in which the value of *V_nS_* depends on the values of *D* and *l* when *f*_0_ = 35 kHz and *p* = 1.

In Figure 6, the sensor internal noise *V_nS_* decreases slowly with the equivalent radius *l*. The red line in Figure 6 represents the sensor internal noise *V_nS_* set as 10 nV/m^2^. Coil diameter *D* and cross-section radius *l* corresponding to the area above the red line satisfy the conditions, which means that a larger diameter *D* with a larger cross-section radius *l* can achieve lower sensor internal noise *V_nS_*. Finally, the cross-section radius of the coil is designed as 23 mm.

#### 4.4.3. Determination of the Bandwidth of the System

**Figure 7:**
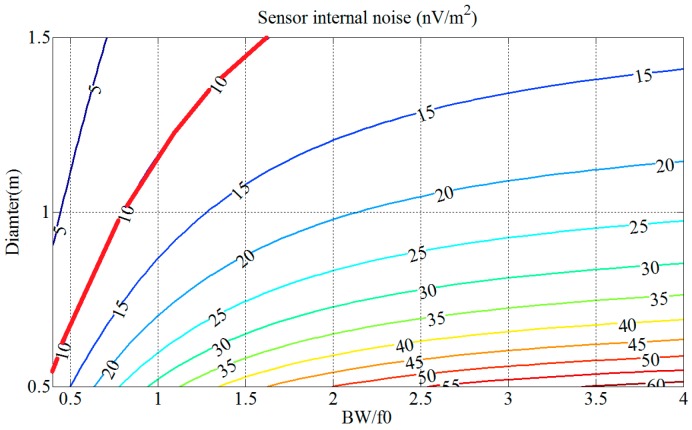
Contour map of the normalized sensor internal noise *V_nS_* versus diameter *D* and ratio of BW to *f*_0_.

By Equation (23), the contour map of the sensor internal noise *V_nS_* is shown in Figure 7, in which the value of *V_nS_* depends on the values of *D* and the ratio of BW to *f*_0_ when *l* = 23 mm and *f*_0_ = 35 kHz.

According to Figure 7, the sensor internal noise *V_nS_* of the sensor increases rapidly with the bandwidth *pf*_0_. The red line in Figure 7 represents the sensor internal noise *V_nS_* set as 10 nV/m^2^. Coil diameter *D* and bandwidth *pf*_0_ corresponding to the area above the red line satisfy the conditions, which means that a larger diameter *D,* with a lower bandwidth *pf*_0_, can achieve lower sensor internal noise *V_nS_*. Generally, the sampling frequency is chosen to be twice the resonant frequency in order to reduce sensor internal noise as much as possible.

Finally, the diameter *D*, resonant frequency *f*_0_, equivalent radius *l*, and bandwidth *pf*_0_ are determined as 1.1 m, 35.6 kHz, 23 mm, and 35.6 kHz, respectively. The detailed parameters of the sensor are given in Section 6.

## 5. Electromagnetic Compatibility (EMC) Design of the Sensor

EMC design is essential for the sensitivity of the sensor, especially for systems working in complex electromagnetic environments. In addition to suppressing the common mode noise by a differential structure sensor, electrical shielding has also been developed to eliminate the electrical noise directly coupled to the sensor. The design of coil shielding will be discussed in detail here.

### 5.1. Effectiveness of Coil Shielding

In general, coupling capacitance exists between conductors or between a conductor and the ground. When the ground is chosen as the reference zero potential, the interference potential of a conductor (also called interference source) will have an impact on other conductors through the coupling capacitors.

**Figure 8:**
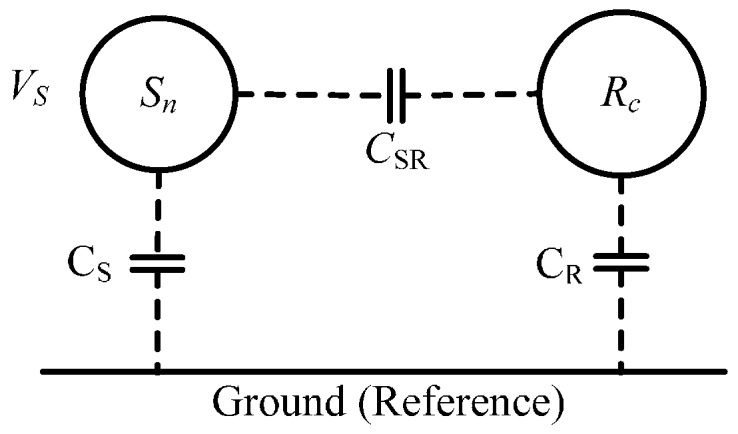
Coupled schematic of electrical interference.

As shown in Figure 8, the coupling capacitors between interference source *S_n_*, conductor *R_c_,* and the ground are *C_S_*, *C_R_*, and *C_SR_*, respectively. If the potential of the interference source *S_n_* to the ground is *V*_S_, the coupled potential *V_R_* of the conductor *R_c_* through the coupling capacitive *C_SR_* and *C_R_* can be expressed as:

(24) $$V_{R}=\frac{V_{S}}{1+C_{R}/C_{SR}}$$

From Equation (25) it can be seen that the interference voltage of the conductor *R_c_* can be suppressed by reducing the coupling capacitance *C_SR_* or increasing the coupling capacitance *C_R_*.

**Figure 9:**
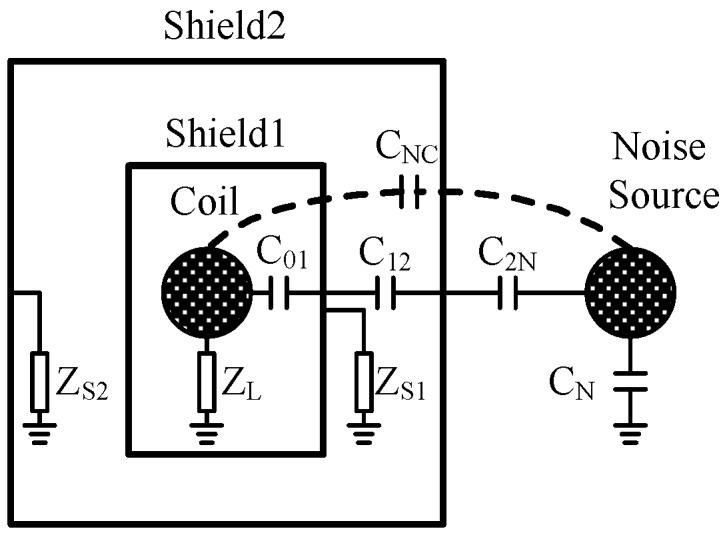
Coupling capacitors of a double shielded coil.

The electrical coupling noise can be effectively suppressed by the electrical shielding. The coupling capacitors of a double-shielded coil are shown in Figure 9.

In Figure 9, *C*_01_, *C*_12_, *C*_2N_, *C*_NC_, and *C*_N_ are coupling capacitors between the coil, shielding, noise source, and the ground. *Z*_L_, *Z*_S1_, and *Z*_S2_ are the grounding impedance of the coil and shielding. Electrical noise of the coil coupled from the interference source through the shield can be described as follows.

**Figure 10:**
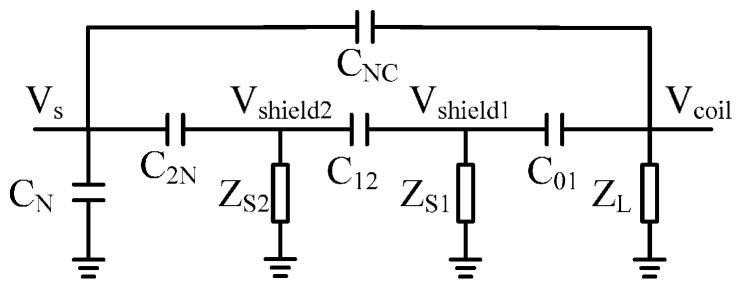
Equivalent circuit of coil shielding.

Usually, the direct coupling capacitance *C*_NC_ between coil and noise source can be neglected. The grounded impedance *Z*_S1_ and *Z*_S2_ are much lower than the impedance of the capacitance 1/*jωC*_12_ and 1/*jωC*_2N_. According to Figure 10, the transmission characteristic of this circuit can be approximated as:

(25) $$\frac{V_{coil}}{V_{S}}\approx \frac{\left(j\omega C_{12}Z_{S1}\right)\left(j\omega C_{2N}Z_{S2}\right)}{1/\left(j\omega Z_{L}C_{01}\right)+1}$$

According to Equation (25), the coupled electrical noise of the coil is proportional to the product of the grounded impedance and the coupling capacitor. Thus, reducing the grounding impedance of the shields can effectively eliminate the coupling noise. In the limit when the grounded impedance tends to zero, *jωC*_12_*Z*_S1_ and *jωC*_2N_*Z*_S2_ tend to zero, too, which means that the greater the number of shielding layers, the lower the coupling noise. Finally, triple-shielding is adopted for the coil to improve the effectiveness of coil shielding.

### 5.2. Structure of Coil Shielding

**Figure 11:**
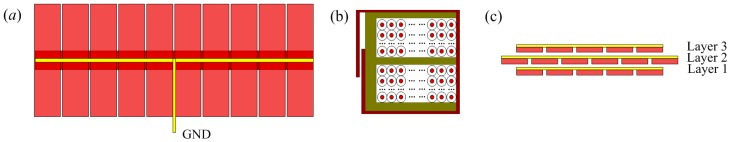
(**a**) Structure of the shielding; (**b**) section of the shielding; and (**c**) relative position of the different layers

As discussed above, a perfect grounding with extremely low impedance is critical to shielding effectiveness. Additionally, the transmitter current is very large and switches off very quickly, resulting in a strong induced current in the shield. This induced current will have a significant influence on the ground response, so it must be suppressed. All of these performances depend on the structure of the shielding, which is proposed in Figure 11.

As shown in Figure 11a, the shielding is composed of a long copper foil connected with a number of short copper foils perpendicular to the long one. The short copper foils around the coil cannot close as a loop, as shown in Figure 11b. This structure can effectively suppress the eddy current but has almost no effect on shielding effectiveness. A copper wire is attached along the long copper foil to enhance the conductivity of the whole shielding. The wire drawn from the center of the shielding is connected to the ground of the preamplifier to minimize the grounding impendence of shielding. When multi-layer shielding is adopted, the shielding of the different layers should be placed as shown in Figure 11c to reduce the direct coupling noise from the interface source.

## 6. Experiment

**Figure 12:**
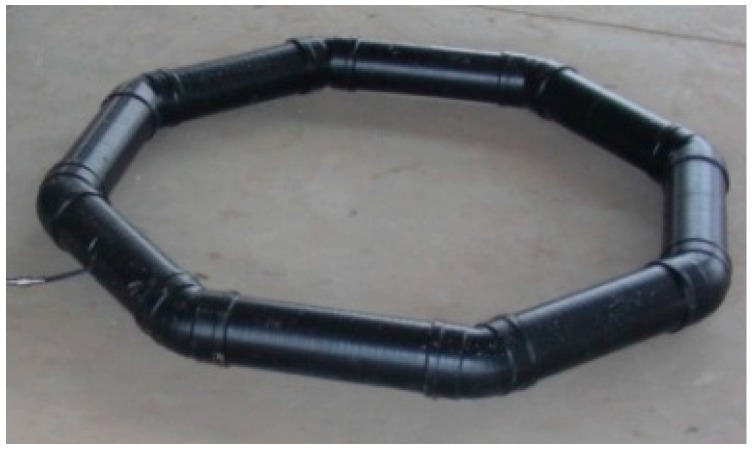
Experimental model of the AEM sensor.

According to the discussion in Section 4 and Section 5, an experimental model of the AEM sensor with a sensor internal noise of 10 nV/m^2^ was designed and constructed, as shown in Figure 12.

The diameter *D*, number of turns *n*, resonant frequency *f*_0_, and the equivalent radius *l* are 1.1 m, 120, 35.6 kHz, 23 mm, respectively. A low-noise operational amplifier LT1028 (Linear Technology Corporation, Milpitas, CA, USA) was chosen to amplify the induced voltage. The specifications of the sensor are listed in Table 2.

### 6.1. Sensor Internal Noise of the Sensor

**Figure 13:**
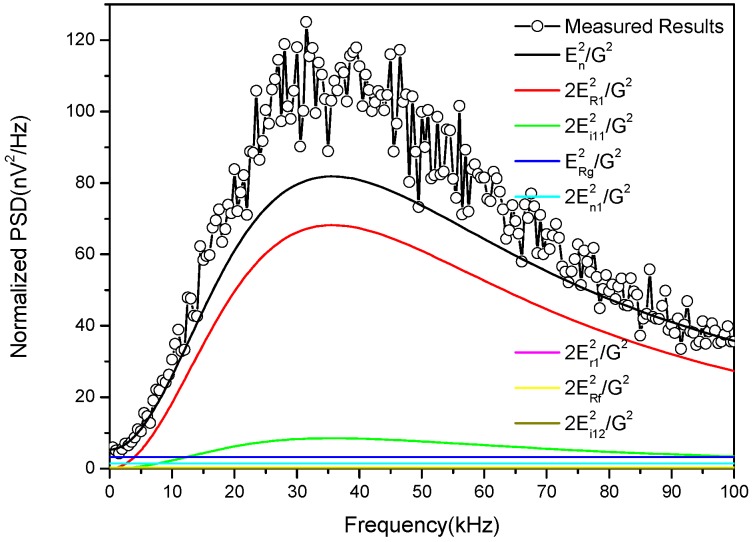
Comparison of calculated and measured PSD of the sensor.

Based on the parameters in Table 2, the normalized power spectrum of different noises in Equation (17) is calculated and compared with the measured PSD of the sensor by an Advantest R9211E digital spectrum analyzer (Advantest, Tokyo, Japan), as shown in Figure 13.

As shown in Figure 13, both the measured and calculated normalized noise PSD of the sensor first increase with frequency and then decrease, reaching a maximum at the resonant frequency. According to the calculated results, the thermal noise of the damping resistor is the main contributor to the sensor internal noise, followed by the current noise of the amplifier. Compared with the thermal noise of the damping resistor, noises of the DC resistor, the gain resistor, the feedback resistor, voltage noise, and the current noise of the amplifier can be neglected.

The calculated results agree well with the experimental data. The maximum value of the measured results is about 110 nV^2^/Hz, while the calculated result gives 80 nV^2^/Hz. The difference between the two comes from the spectrum analyzer and electromagnetic interference that may not be completely eliminated by the shielding room. According to Equation (20), the sensor internal noise is calculated as 13.3 nV/m^2^, 33% higher than the theoretical value.

### 6.2. The Shielding Effectiveness Testing

**Figure 14:**
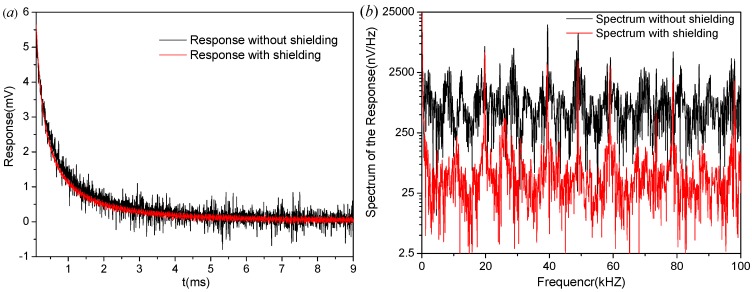
(**a**) Shielding effectiveness testing in time domain; and (**b**) shielding effectiveness testing in frequency domain.

Shielding effectiveness of the sensor is tested under an extremely harsh electromagnetic environment. First, the sensor works with triple-shielding connected to the ground of the pre-amplifier. Then, the shielding is removed and a pair of external capacitors is connected in parallel to the input of the pre-amplifier to maintain a constant resonant frequency. The shielded coil shows a much lower noise level, both in time and frequency domains, as shown in Figure 14.

In Figure 14a, the coupled electrical interference appears as a series of pulses directly superimposed on the signal. The shielding can suppress the electrical interference significantly. The peak-to-peak noise of the unshielded sensor is approximately 1.5 mV, while for the shielded case it is 0.3 mV, which is only 20% of the unshielded value. Compared to the unshielded response, the noise spectrum of the shielded response decreases more than one order of magnitude, which means an improvement of more than 20 dB in the SNR for the coupled interference, as shown in Figure 14b. Thus, shielding can greatly suppress the electrically-coupled noise of the sensor, especially in harsh electromagnetic environments.

## 7. Conclusions and Prospects

An improved high-sensitivity differential transient electromagnetic sensor with triple-shielding for the AEM system was designed, built, and tested to reduce the sensor internal noise and to improve shielding effectiveness in exploration surveys. With the sensor internal noise level determined as 10 nV/m^2^, the design and optimization of the sensor was described in detail.

The sensor internal noise model with a damping resistor and a preamplifier was established and analyzed. The results showed that the thermal noise of the damping resistor dominates the sensor internal noise. To realize a high-sensitivity sensor, the diameter of the coil should be as large as possible and the resonant frequency should be as small as possible. Decreasing the bandwidth of the system is an effective way to obtain high sensitivity. However, Nyquist sampling bandwidth must be guaranteed to prevent signal distortion. The section size has little effect on the sensor internal noise.

A multi-layer electromagnetic shielding system with center grounding for a TEM sensor was developed and analyzed. To improve shielding effectiveness of the sensor, the number of the shielding layers should be as high as possible and the impedance of the shield grounding should be as low as possible. Multi-layered shielding with center grounding was adopted in our study. Additionally, unclosed copper foils connected by a wire are also adopted to eliminate the eddy currents.

Finally, the sensor was constructed with a diameter, resonant frequency, and normalized equivalent input noise of 1.1 m, 35.6 kHz, and 13.3 nV/m^2^, respectively. Experiments were conducted to verify the sensor internal noise and shielding effectiveness of the sensor. The conformity between the experimental and simulation results confirmed the theory of sensor internal noise model of the sensor. Shielding effectiveness of over 20 dB was achieved in a complex electromagnetic environment, which reflects a good electromagnetic compatibility of the sensor.

The sensor described in this paper was specially designed for deep penetration in an AEM system. The noise induced in the sensor could be greatly suppressed for a low resonant frequency. The noise directly coupled to the sensor could also be effectively suppressed by the multi-layer shielding. For these advantages, the sensor described here is of great significance to deep penetration in AEM systems.

## Figures and Tables

**Table 1:** Normalized power spectral density (PSD) for all types of noise sources.

Noise Sources	Range (*m*: 1–9)	Normalized PSD (nV^2^/Hz)
Dc resistance *r*_1_ (Ω)	*m*~*m ×* 10	16*m ×* (0.001~0.01)
Damping resistance *R*_1_ (kΩ)	*m*	16*m*
Gain resistance *R_g_* (Ω)	*m*~*m ×* 10	16*m ×* (0.001~0.01)
Feedback resistance *R_f_*_1_ (kΩ)	*m*	16*m*/G^2^
Voltage noise of amplifier *e_n_* (nV/$\sqrt{\mathrm{Hz}}$)	*m*	*m*^2^
Current noise of amplifier *i_n_*_11_ (pA/$\sqrt{\mathrm{Hz}}$)	<1	<*m*^2^
Current noise of amplifier *i_n_*_12_ (pA/$\sqrt{\mathrm{Hz}}$)	<1	<*m*^2^/G^2^

**Table 2:** Fabricated parameters of AEM sensor.

Parameters	Symbol	Value
Equivalent diameter of the coil	*D*	110 cm
Number of turns	*n*	120
Dimensions of the coil section	*a*, *b*	40 mm, 40 mm
Area of the copper	*S*_c_	0.4 mm^2^
Inductance of the coil	*L*_1_ *= L*_2_	18.5 mH
DC resistance of the coil	*r*_1_ *= r*_2_	9.2 Ω
Capacitance of the coil	*C*_1_ *= C*_2_	1080 pF
Resonant frequency of the coil	*f*_0_	35.6 kHz
Resistor	*R*_1_ *= R*_2_, *R_g_*, *R_f_*_1_ = *R_f_*_2_	2070 Ω, 100 Ω, 500 Ω
Voltage noise of the amplifier	*e_n_*_1_ = *e_n_*_2_	0.85 nV/$\sqrt{\mathrm{Hz}}$
Current noise of the amplifier	*i_n_*_11_ = *i_n_*_12_ = *i_n_*_21_ = *i_n_*_22_	1.0 pA/$\sqrt{\mathrm{Hz}}$
